# Allosteric modulators of solute carrier function: a theoretical framework

**DOI:** 10.3389/fphys.2023.1166450

**Published:** 2023-05-05

**Authors:** D. Boytsov, K. Schicker, E. Hellsberg, M. Freissmuth, W. Sandtner

**Affiliations:** ^1^ Center of Physiology and Pharmacology, Medical University of Vienna, Vienna, Austria; ^2^ Computational Structural Biology Unit, National Institute of Neurological Disorders and Stroke (NINDS), National Institutes of Health (NIH), Bethesda, MD, United States

**Keywords:** transition-state theory, linear free energy relationship, allosteric modulator, drug design, solute carrier

## Abstract

Large-scale drug screening is currently the basis for the identification of new chemical entities. This is a rather laborious approach, because a large number of compounds must be tested to cover the chemical space in an unbiased fashion. However, the structures of targetable proteins have become increasingly available. Thus, a new era has arguably been ushered in with the advent of methods, which allow for structure-based docking campaigns (i.e., virtual screens). Solute carriers (SLCs) are among the most promising drug targets. This claim is substantiated by the fact that a large fraction of the 400 solute carrier genes is associated with human diseases. The ability to dock large ligand libraries into selected structures of solute carriers has set the stage for rational drug design. In the present study, we show that these structure-based approaches can be refined by taking into account how solute carriers operate. We specifically address the feasibility of targeting solute carriers with allosteric modulators, because their actions differ fundamentally from those of ligands, which bind to the substrate binding site. For the pertinent analysis we used transition state theory in conjunction with the linear free energy relationship (LFER). These provide the theoretical framework to understand how allosteric modulators affect solute carrier function.

## Introduction

Biological membranes are diffusion barriers for polar solutes. The solute carrier (SLC) group of membrane proteins affords the permeation of polar solutes across plasma- and intracellular membranes (Colas et al., 2016). Accordingly, SLCs play a central role in maintaining cell homeostasis, in supporting metabolism and in extruding toxic compounds (Klaassen and Aleksunes, 2010; Kristensen et al., 2011; Zhang et al., 2019). Given their physiological importance, it is not surprising that many inherited diseases have been linked to mutations in SLC genes (Bhat et al., 2021): in fact, of 9,178 monogenic diseases listed in the OMIM database, 187 and 434 are linked to SLCs and transporters, respectively. More importantly, SLCs can be plausibly argued to be druggable targets, which are underrepresented when compared to other protein families (Wang et al., 2020). Progress in identifying new SLC ligands, however, is slow (Casiraghi et al., 2021). Therefore, there is an urgent need to develop new strategies in drug development.

Historically, the majority of therapeutically relevant drugs were found by serendipity. Some 30 years ago, large-scale drug screens (high-throughput screening) were introduced to probe the chemical space for ligands with suitable properties. High-throughput screening has several limitations, though, in particular false positives and false negatives, which can result in substantial costs (Mayr and Bojanic, 2009). The structures of candidate drug targets are being solved at an increasing pace. Accordingly, approaches based on bioinformatics have become an attractive alternative, because they can be used to conduct virtual screens. Structure-based docking of large chemical libraries has resulted in the discovery of novel ligands for e.g., several G protein-coupled receptors (Orry et al., 2006; Kolb et al., 2009; Kolb and Irwin, 2009; Katritch et al., 2011; Rognan, 2011; Gabrielsen et al., 2012; Kufareva et al., 2012; Gabrielsen et al., 2013; Ngo et al., 2016; Rognan, 2017; Roth et al., 2017; Scharf et al., 2019; Alon et al., 2021; Ballante et al., 2021; Bender et al., 2021); in addition, the σ2-receptor is another recent example (Alon et al., 2021). Transporters can also be targeted by this approach: Singh et al. (2022) provided a proof-of-concept by docking a large library against the inward-facing conformation of the serotonin transporter (SERT), which led to the identification of molecules of novel chemotypes and pharmacology.

The monoamine transporters—i.e., the transporters for norepinephrine (NET/SLC6A2), dopamine (DAT/SLC6A3) and serotonin (SERT/SLC6A4) stand out among the SLC family members: they have a rich pharmacology (Sitte and Freissmuth, 2015). Most drugs bind to the orthosteric binding site (i.e., the substrate binding site). However, some compounds were reported to bind to allosteric sites (e.g., vilazodone, ATM7) (Kortagere et al., 2013; Plenge et al., 2021). Allosteric modulators are of interest because they can exert actions, which orthosteric ligands do not: this includes a drug-induced acceleration of the transport cycle, increased/decreased substrate release and allosteric (i.e., non-competitive) inhibition of substrate uptake (Hasenhuetl et al., 2019; Niello et al., 2020). Arguably, any of these actions may prove to be useful from a therapeutic perspective. However, at the current state of affairs, there isn’t any theoretical framework for the rational design of allosteric modulators. This framework must take into account how SLCs operate. Only then can we identify the key levers for modulating SLC function by small molecule modulators. Substrate uptake by solute carriers is contingent on their ability to undergo a series of partial reactions. These include binding/unbinding reactions of (co)-substrates and conformational rearrangements such as the isomerization from the outward-facing (OF) to the inward-facing (IF) state (Rudnick and Sandtner, 2019). The collective partial reactions form a closed loop, which is also referred to as the transport cycle. In the present study, we used transition state theory and the linear free energy relationship (LFER) to analyze and predict the effects of allosteric modulators on the reaction kinetics of solute carriers.

## Methods

**FIGURE 1:**
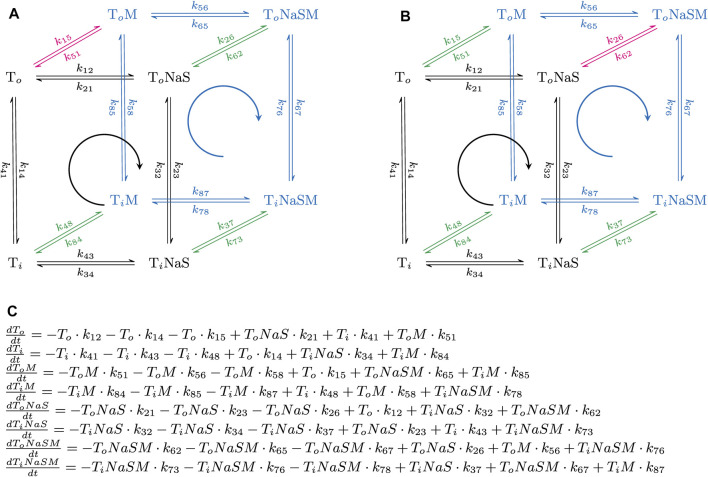
Simplified reaction schemes of the transport cycle of a solute carrier in the absence and presence of an allosteric modulator. **(A,B)** show the reaction diagrams of the kinetic models, which were used to emulate the action of a positive and a negative allosteric modulator, respectively. **To** and **Ti** are the substrate-free outward (OF)- and substrate-free inward-facing (IF) conformations. **ToNaS** is the (co)-substrate bound OF state and **TiNaS** the (co)-substrate bound IF state. **ToM**, **TiM**, **ToNaSM** and **TiNaSM** are the modulator-bound counterparts. The reaction diagrams are comprised of one reaction loop in the front (indicated in black) and one in the rear (indicated in blue). The two loops, which represent the transport cycle of a hypothetical solute carrier in the absence (front loop) and presence (rear loop) of the modulator (M), were biased in the forward direction (i.e., clockwise) by setting the concentration of Na^+^ to 150 mM on the extracellular and to 0 mM on the intracellular side. The red and green arrows indicate the high and low affinity binding reaction(s) of the modulator, respectively. In **(A)**, the modulator was assumed to bind with higher affinity to the **To** state. In **(B)**, the modulator was assumed to bind with higher affinity to the **ToNaS** state. **(C)** System of differential equations, which underlies the reaction schemes in **(A,B)**. Each of the displayed equations defines the time-dependent change in state occupancy of one of the eight states specified in **(A,B)**.

Figure 1 displays the reaction scheme of the model used to simulate the effect of positive (Figure 1A) and negative allosteric modulators (Figure 1B) on the transport cycle of a solute carrier. The schemes depicted in Figures 4A, 5A are simplified representations thereof. In Figure 1C we show the system of differential equations, which underlies the two reaction schemes. The shown reaction diagrams are comprised of one reaction loop in the front (indicated in black) and one in the rear (indicated in blue). The two loops, which represent the transport cycle of a hypothetical solute carrier in the absence and presence of the modulator, were biased in the forward direction (i.e., clockwise) by setting the concentration of Na^+^ to 150 mM on the extracellular and to 0 mM on the intracellular side. In the absence of the modulator all transporters cycle in the front loop, while at saturating concentrations of the modulator all transporters cycle in the rear loop. At concentrations below saturation, the fraction of the transporter, which is free and occupied by the modulator, cycles in the front and rear loop, respectively. Positive allosteric modulation of substrate transport was modelled by assuming that the substrate-free outward-facing state (state **To** in the diagram) had a higher affinity for the modulator than all other states in the diagram. Negative allosteric modulation of substrate transport was modelled by assigning high affinity of the modulator to the (co)-substrate-bound outward-facing conformation (state **ToNaS** in the diagram). In the model, high affinity was conferred to an allosteric ligand by lowering its dissociation rate.

The following set of rate constants, which we used to parameterize the reaction scheme shown in Figure 1A (i.e., for a positive allosteric modulator), is listed here as an example: k_12_ = 10^16^*[Na_out_]*[S_out_]s^−2^, k_23_ = 10^6^ s^−1^, k_34_ = 10^12^ s^−2^; k_41_ = 2 s^−1^ (i.e., rate constants in the clockwise direction of the front loop); k_14_ = 2 s^−1^; k_43_ = 10^16^*[Na_in_]*[S_in_] s^−2^; k_32_ = 10^6^ s^−1^; k_21_ = 10^12^ s^−2^ (i.e., rate constants in the counter-clockwise direction of the front loop); k_56_ = 10^16^*[Na_out_]*[S_out_]s^−2^; k_67_ = 10^6^ s^−1^; k_78_ = 10^12^ s^−2^; k_85_ = 20 s^−1^*X (i.e., rate constants in the clockwise direction of the rear loop); k_58_ = 2 s^−1^*X; k_87_ = 10^16^*[Na_in_]*[S_in_]s^−2^; k_76_ = 10^6^ s^−1^; k_65_ = 10^13^ s^−2^ (i.e., rate constants in the counter clockwise direction of the rear loop); k_26_ = 10^6^*[M]*s^−1^; k_62_ = 10 s^−1^; k_37_ = 10^6^*[M]*s^−1^; k_73_ = 10 s^−1^; k_48_ = 10^6^*[M]*s^−1^; k_84_ = 10 s^−1^ (i.e., rate constants, which define binding of the modulator to the low affinity states); and k_15_ = 10^6^*[M]s^−1^; k_51_ = 1 s^−1^ (i.e., rate constants, which define binding of the modulator to the high affinity state). [Na_out_] and [S_out_] are the extracellular Na^+^ and substrate concentration, respectively, [Na_in_] and [S_in_] the corresponding intracellular concentrations, and [M] the concentration of the allosteric modulator. **X** is a factor that allows for adjusting the value of *α* (i.e., the position of the transition state on the reaction coordinate). **X** can assume values between zero and infinity. The loop was biased into the forward direction by setting [Na_out_], [S_out_], [Na_in_], and [S_in_] to 0.15 M, 10^−3^ M, 0 M and, 0 M, respectively. The chosen set of rate constants (i) keeps microscopic reversibility, (ii) sets the K_D_s of the modulator for the low and high affinity state of the hypothetical transporter to 10 and 1 µM, respectively and (iii) allows for the reaction from TiM to ToM to remain rate-limiting over a wide range of **X** values.

We used the Systems Biology Toolbox (Schmidt and Jirstrand, 2006) and MATLAB 2018a (MathWorks, Natick, MA, United States) to evaluate by numerical integration the time-dependent changes in state occupancies of the system of differential equations shown in Figure 1C. Substrate uptake was modelled as the sum of the flux through the front and the rear loop.

We used the Eyring equation to relate the unidirectional rate constants of a reaction to the free energy of the corresponding transition state ( $G^{‡}$ ).

$$k=\kappa *\frac{k_{B}}{h}*T*e^{\frac{-∆G^{‡}}{RT}}$$

where k is the unidirectional rate constant, k_B_/h (Boltzmann constant/Plank constant) is the universal attempt frequency, T is the temperature and R is the gas constant. For the transmission coefficient κ we assumed a value of 1 (i.e., no reflection from the transition state back to the reactant state). We calculated the position of the transition state on the reaction coordinate (i.e., *α*) by converting the values of the unidirectional rate constants into the corresponding values of $G^{‡}$ and used these to construct the free energy landscape. The following equation allowed for extracting *α*: $\alpha ={\left(∆∆G\right.}^{‡}-∆{∆G}_{R})/\left(∆∆G_{P}+∆{∆G}_{R}\right)$ . The energy terms in this equation are illustrated in Figure 3.

For the molecular visualization in Figure 7 we used PyMOL (The PyMOL Molecular Graphics System, Version 2.5 Schrödinger, LLC) (Schrodinger, 2022).

## Results

### Allosteric modulators of SLC function

**FIGURE 2:**
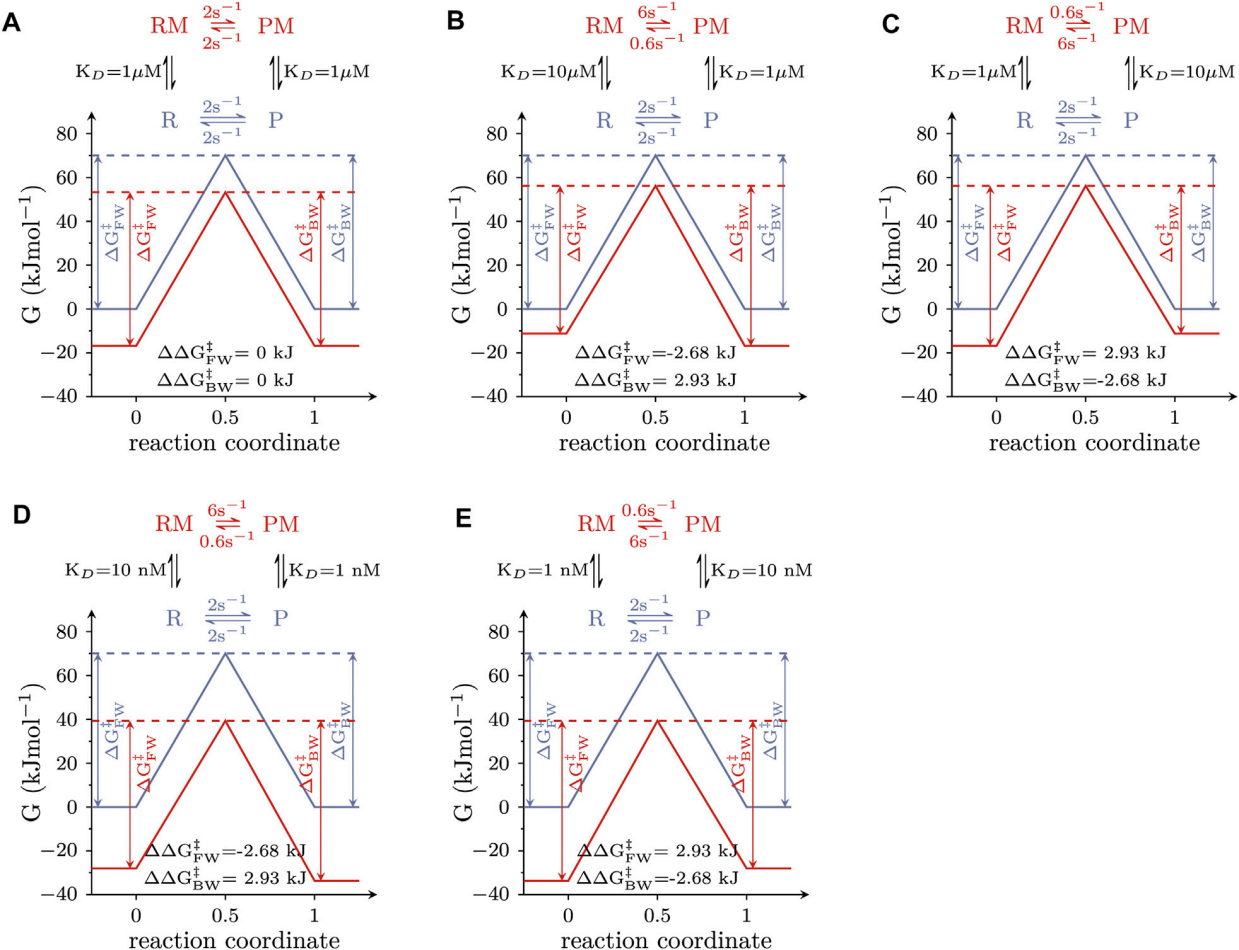
Free energy landscapes of elementary reactions that are responsive to modulation by a ligand. The blue and red lines show the landscape of the Gibbs energy (G) in the absence and presence of 1 mM modulator, respectively. The corresponding reaction scheme is displayed on the top of each panel, where R and P are the reactant and the product state, and RM and PM are the modulator-bound counterparts. **(A)** Free energy landscapes of a modulator that binds to both states with equal affinity (i.e., K_D_ = 1 µM). Stated in the figure are the values of ∆∆ $G_{FW}^{‡}$ and ∆∆ $G_{FW}^{‡}$ . **(B)** The same as in **(A)** for an allosteric modulator that binds to the product state with higher affinity than the reactant state (i.e., K_D_ = 1 µM and 10 μM, respectively). **(C)** The same as in **(A)** for an allosteric modulator that binds the reactant state with higher affinity than the product state (i.e., K_D_ = 1 µM and 10 μM, respectively). The position of the transition state in **(A–C)** was assumed to be located halfway on the reaction coordinate. **(D,E)** Free energy landscapes of a hypothetical allosteric modulator, which binds to the reactant and product state with affinities in the nanomolar range. In **(D)** the K_D_s for the reactant and product state are 10 and 1 nM, respectively. In **(E)** the K_D_s for the reactant and product state are 1 and 10 nM. The affinity ratio (i.e., K_D_ reactant state/K_D_ product state) is 10 in **(B,D)** and 0.1 in **(C,E)**. As a consequence, the reaction rates in **(B)** are the same as in **(D)** and the reaction rates in **(C)** the same as in **(E)**.

Allosteric modulators of solute carriers are molecules, which act by changing the rate(s) of one or more partial reactions in the transport cycle of the targeted transporter. For a molecule/ligand to alter the rate of a partial reaction, it must bind with higher affinity to the reactant than to the product state or *vice versa*. This point is illustrated in Figure 2, which shows the free energy landscapes of a reversible elementary reaction that is amenable to modulation by a ligand. The blue and red lines represent the free energy landscapes in the absence and presence of an allosteric modulator, respectively. The insets depict the corresponding reaction schemes, where the modulator-free reactant and product-state are indicated as R and P and the modulator-bound counterparts as RM and PM. In the absence of the allosteric modulator, the reaction rate in the forward direction was assumed to equal the rate in the backward direction. We considered three scenarios: (i) The allosteric modulator binds to the reactant and the product state with equal affinity (Figure 2A). A modulator with these properties reduces both, the free energy (G) of the reactant and the product state by the same amount. Thus, ∆ $G^{‡}$ (the free energy difference between the ground- and the transition state) in the forward (∆ $G_{FW}^{‡}$ ) and the backward direction (∆ $G_{BW}^{‡}$ ) remains constant (compare the length of the red and blue arrows in Figure 2A). The Eyring equation (see Methods) allows for converting ∆ $G_{FW}^{‡}$ and ∆ $G_{BW}^{‡}$ into the unidirectional rate constants in the forward and backward direction, respectively. Because ∆ $G_{FW}^{‡}$ and ∆ $G_{BW}^{‡}$ are unchanged the rate constants of this reaction (values in blue) are not affected by the allosteric modulator. (ii) The allosteric modulator binds to the product state with a tenfold higher affinity than to the reactant state (Figure 2B). In this case, the modulator lowers the free energy of the product state more than that of the reactant state. As a result, ∆ $G_{FW}^{‡}$ becomes smaller and ∆ $G_{BW}^{‡}$ larger. This leads to an increase and decrease of the unidirectional rate constant in the forward and backward reaction, respectively (see reaction diagram Figure 2B). (iii) The allosteric modulator binds to the reactant state with higher affinity than to the product state (Figure 2C). In this instance, the rate constant in the forward direction decreases while the rate constant in the backward direction increases. Thus, depending on which state the allosteric modulator favors, a reaction proceeding in the forward direction can either be slowed or accelerated. In Figures 2A–C we assumed that the allosteric modulator bound to the reactant and the product state with affinities in the micromolar range. In Figures 2D, E we show the free energy landscapes, in the presence and absence of a modulator with K_D_s in the low nanomolar range. In Figure 2D the K_D_ is 10 and 1 nM for the reactant and product state, respectively, and the reverse in Figure 2E. The low and high affinity modulators affect the rates of the reaction to the same extent (cf., 2B and 2D; cf., 2C and 2E), provided that the affinity ratios for reactant and product state (K_D_ reactant state/K_D_ product state) are equivalent. This demonstrates that it is not the absolute but the relative affinity of the modulator for the two ground states, which determines the extent of the change in the reaction rate.

The reaction schemes in Figure 2 comprise a loop. The rules of thermodynamics dictate that the numeric values of the rate constants used to parameterize a reaction loop must fulfill the detailed balance constraint (the product of the rates in the forward direction of the loop must equal the product of the rates in the backward direction). This constraint explains why in the reaction schemes in Figures 2B–E the rate constants in the forward and the backward direction, connecting the RM and PM state differ by a factor of 10: it results from the tenfold difference in the K_Ds_ of the allosteric modulator for R and P (and from the requirement for maintaining microscopic reversibility). However, it is evident that other pairs of rate constants exist, which also fulfill the detailed balance constraint (e.g., 8 and 0.8 s^−1^). In the following, we show that the specific values stated in the reaction schemes in Figures 2B–E are dictated by the linear free energy relation.

### Linear free energy relationship

LFER links the change in ∆G of the reactant and product state (i.e., $∆{∆G}_{R}$ ; $∆∆G_{P}$ ) to the corresponding change of ${∆G}^{‡}$ (i.e., ∆∆ $G^{‡}$ ). When LFER applies, these parameters are related as follows:

$${∆∆G}^{‡}=\alpha *∆∆G_{P}+\left(1-\alpha \right)*∆{∆G}_{R}$$

**FIGURE 3:**
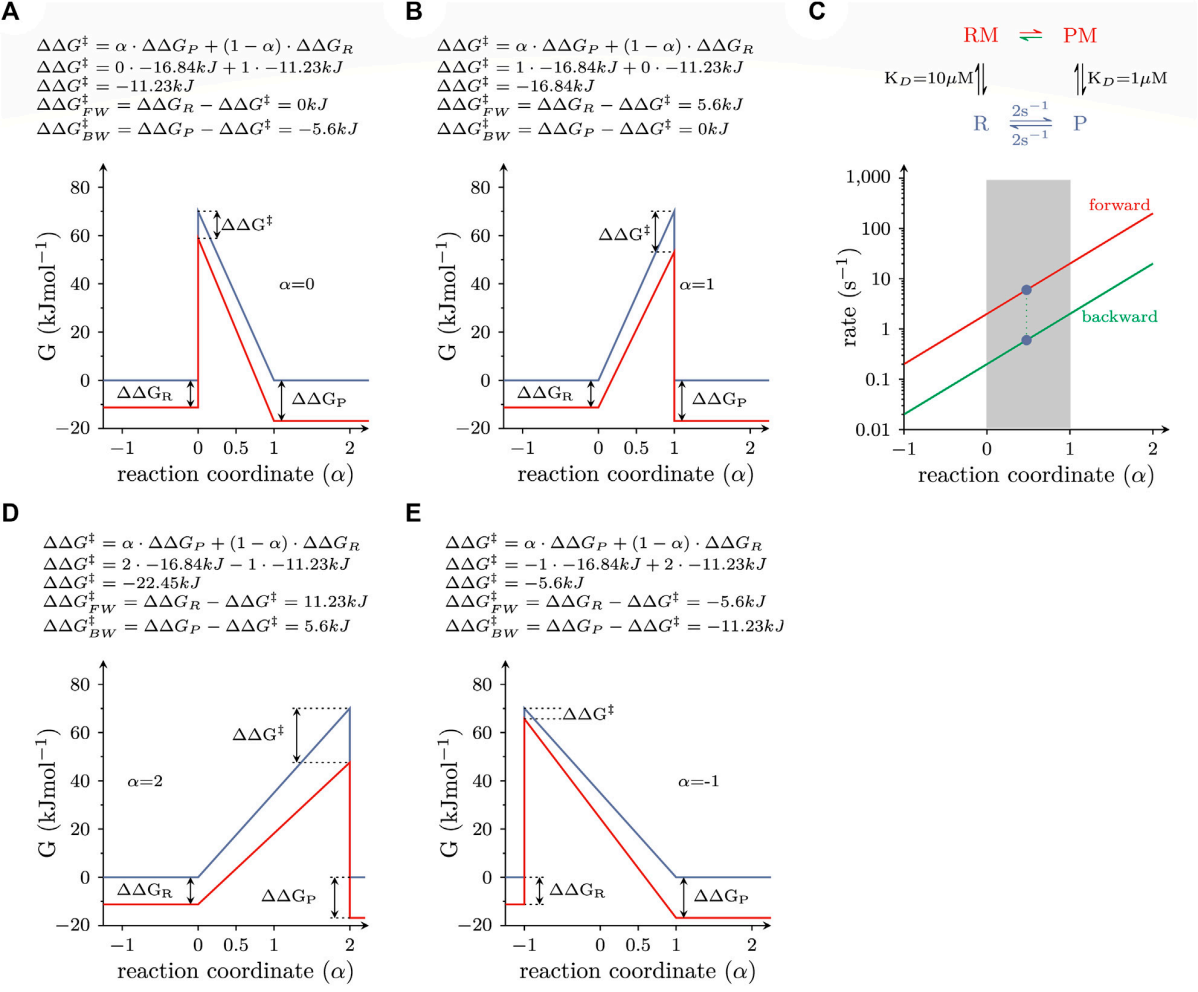
Energy landscapes of reactions, which do or do not comply with LFER **(A)** Free energy landscape of a reversible elementary reaction, which is responsive to modulation by a ligand. The blue and red lines are the landscapes in the absence and presence of 1 mM ligand, respectively. The equation on top of the panel provides a mathematical description of the LFER principle. For the calculation, *α* was assumed to equal zero (i.e., the properties of the transition state resemble those of the reactant state). **(B)** Same as in **(A)**, but with the assumption that *α* equals 1 (i.e., the properties of the transition state resemble those of the product state). **(C)** Plotted are the rate constants connecting the RM and PM states, which fulfill the detailed balance constraint as a function of *α*. The rate constants are plotted on a logarithmic axis. The red and green lines show the forward and backward rate constants, respectively. Each pair of rate constants on a vertical line defines a unique free energy landscape. Pairs that fall into the shaded range comply with LFER. All other pairs do not. **(D,E)** show the free energy landscapes of two pairs of rate constants, which defy LFER. In D and E, *α* is 2 and −1, respectively.

The energy terms of the equation are illustrated in Figure 3. The above equation implies that the transition state of a reaction must have properties, which lie between those of the reactant and the product state (i.e., the ground states). For instance, if a chemical reaction is accompanied by a volume change, the transition state is posited to adopt a volume, which lies between that of the reactant and the product. Similarly, the possible positions of the transition state within the energy landscape are constraint: in LFER the transition state is defined by boundaries imposed by the ground states, where the value of $∆∆G^{‡}$ lies between that of $∆∆G_{P}$ and $∆{∆G}_{R}$ . This applies, if the variable *α* in the equation is only allowed to adopt values between 0 and 1. The latter is a key assumption of LFER, where *α* is thought to account for the position of the transition state along the reaction coordinate: *α* equals zero, if the transition state resembles the reactant state. Conversely, *α* equals 1, if the transition state resembles the product state.

The energy landscapes shown in Figure 2 were calculated assuming that the transition state was located halfway on the reaction coordinate of the reaction (i.e., symmetric barrier assumption): accordingly, the properties of the transition state resemble those of both ground states to the same extent. This, however, need not be the case, as the transition state can either resemble more the reactant or the product state.

In the examples depicted in Figure 3, we considered the same scenario as in Figure 2B, i.e., the allosteric modulator bound with 10-fold higher affinity to the product than to the reactant state (K_D_ = 1 vs. 10 µM) and was present at a saturating concentration (1 mM). In Figure 3A, the transition state was assumed to resemble the reactant state (*α* = 0). The change in ∆ $G_{FW}^{‡}$ (i.e., ∆∆ $G_{FW}^{‡}$ ) can be calculated by subtracting ∆∆ $G^{‡}$ from $∆{∆G}_{R}$ . Similarly, the change in ∆ $G_{BW}^{‡}$ (i.e., ∆∆ $G_{BW}^{‡}$ ) can be computed by subtracting ∆∆ $G^{‡}$ from $∆∆G_{P}$ . Under the assumption that *α* is zero the calculated values for ∆∆ $G_{FW}^{‡}$ and ∆∆ $G_{BW}^{‡}$ are 0 and −5.6 kJ/mol, respectively. Thus, ∆ $G_{FW}^{‡}$ remains unchanged, while ∆ $G_{BW}^{‡}$ is increased by 5.6 kJ/mol. As a consequence, the unidirectional rate constant in the forward direction (as calculated by the Eyring equation) is not affected, but the unidirectional rate constant in the backward direction decreases from 2 to 0.2 s^−1^. Figure 3B demonstrates the other extreme, where the transition state resembles the product state (*α* = 1). In this instance, ligand binding lowers $∆G_{FW}^{‡}$ by about 5.6 kJ/mol but ∆ $G_{BW}^{‡}$ is unchanged. As a corollary, the rate in the forward direction increases tenfold, while the rate in the backward reaction remains unchanged. Figure 3C illustrates, how the position of the transition state on the reaction coordinate (*α*) affects the forward and backward rates (red and green line, respectively): we specifically highlight the pair of values (green dots in Figure 3C), which was used to calculate the free energy landscape displayed in Figure 2B (*α* = 0.5), and stress that, in this plot, each pair of rate constants on a vertical line fulfills the detailed balance constraint. We further emphasize that only those pairs of rate constants, which fall into the shaded range, comply with LFER: all other pairs outside of this range also fulfill the detailed balance constraint, but they violate LFER, because *α* adopts values, which are either smaller than zero or larger than 1. Figures 3D, E exemplify these free energy landscapes, which defy LFER. If *α* is assumed to be 2 (Figure 3D), ∆∆ $G^{‡}$ is larger than $∆∆G_{P}$ and $∆{∆G}_{R}$ . Thus, the transition state is more sensitive to the ligand-induced change in ∆G than either of the ground states. The opposite (i.e. ∆∆ $G^{‡}$ < $∆∆G_{P}$ ; ∆∆ $G^{‡}$ < $∆{∆G}_{R}$ ) is true with *α* = −1 (Figure 3E).

### Positive allosteric modulators

**FIGURE 4:**
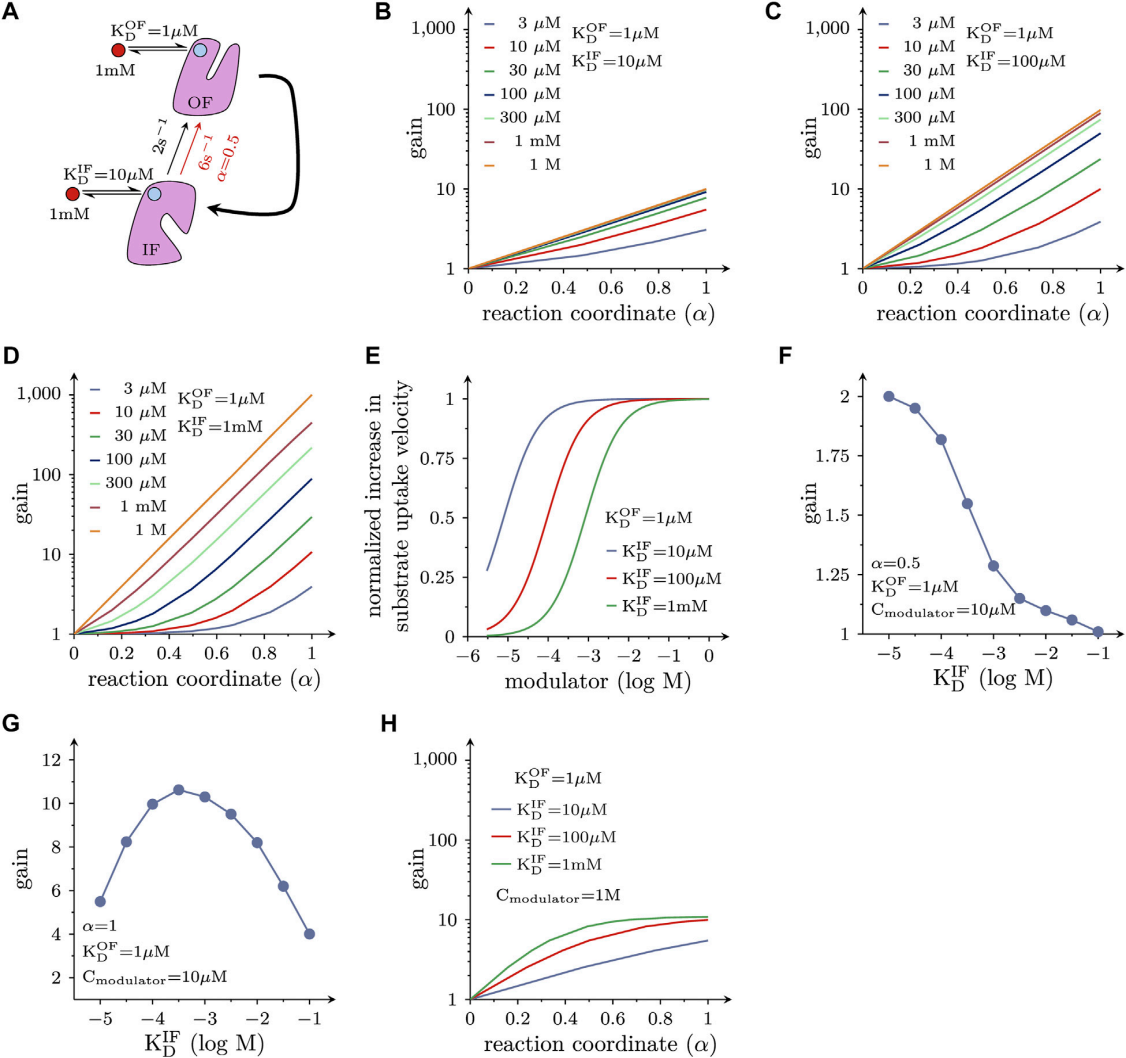
Positive allosteric modulation of solute carrier function. **(A)** Reaction diagram. Displayed is the return step from the substrate-free IF to the substrate-free OF state. This is the rate-limiting reaction in the transport cycle of many, if not most, solute carriers. This reaction was assumed to be a partial reaction in a forwardly biased reaction loop (i.e., in the transport cycle). All other reactions in the loop were lumped together and are represented by the bold curved arrow. The red circle indicates the hypothetical allosteric modulator. The light blue circles indicate the corresponding binding site on the OF state and IF state of the transporter, respectively. In the assumed presence of the modulator the rate of the reaction increased threefold when *α* = 0.5 (i.e., from 2 s^−1^ - black arrow - to 6 s^−1^- red arrow) **(B)** Plotted is the fold increase (gain) in substrate uptake velocity, which is induced by the hypothetical allosteric modulator, as a function of *α*. The modulator was assumed to bind with a tenfold higher affinity to the OF state than to the IF state. The colored lines show the gain at the indicated concentrations of the hypothetical allosteric modulator. If *α* = 0, the modulator is ineffective. **(C,D)** The same type of plots as in **(B)** for a hypothetical allosteric modulator, which is 100-fold and 1000-fold more selective for the OF state, respectively. **(E)** Shown is the normalized increase in the substrate uptake rate as a function of the modulator concentration for three hypothetical modulators with variable selectivity. As seen, the EC_50_ value for the allosteric effect shifts to the right as the modulator becomes more selective for the OF state. **(F)** Gain induced by hypothetical allosteric modulators with variable selectivity for the OF over the IF state (ranging from 10- to 10,000 fold) for *α* = 0.5. At 10 µM of the modulator, the more selective drugs became less efficient. **(G)** Gain as a function of the selectivity of the hypothetical allosteric modulators for a transporter operating at *α* = 1. At 10 µM of the modulator the relation between selectivity and the gain in substrate uptake is bell-shaped. **(H)** Effect of modulators on the transport cycle under the assumption that the slowest reaction in the transport cycle (i.e., 2 s^−1^) is accelerated to an extent that another reaction becomes rate limiting (20 s^−1^): the gain (in substrate uptake velocity) is plotted as a function of *α* for three hypothetical allosteric modulators with differing selectivity for the OF state (i.e., 10-, 100- and 1000-fold). In this instance, the attainable gain is limited, although the modulators are present at a saturating concentration.

Allosteric modulators of SLC function are of interest because they can exert actions other than those elicited by orthosteric ligands (i.e., compounds trapped in the substrate binding site): drug-induced acceleration of the transport cycle is of particular interest, because it leads to increased substrate uptake. For a molecule to increase substrate uptake, it must accelerate the velocity of one or more partial reactions in the transport cycle of the targeted solute carrier. However, a substantial increase in the substrate uptake rate of a transporter, can only be achieved by augmenting the velocity of reactions, which are slow and thus rate-limiting in the transport cycle. For many solute carriers, including the monoamine transporters, the slowest reaction in the transport cycle is the return step from the substrate-free inward-facing (IF) to the substrate-free outward-facing (OF) conformation. This reaction is depicted in the schematic representation in Figure 4A. We simulated cellular substrate uptake through an SLC by focusing on this reaction in the transport cycle, which was biased into the forward direction by an inwardly-directed Na^+^ gradient. All other reactions were lumped together and represented by the curved arrow. It was further assumed that the allosteric ligand was present at a saturating concentration (i.e., 1 mM). Because the cycle is biased in the forward direction, the OF state is the product state. As discussed above, a ligand, which accelerates a reaction, must bind to the product state with higher affinity than the reactant state. Therefore, by analogy with Figures 2B, 3, the ligand affinity for the OF state was assumed to be ten times higher than that of the IF state. LFER predicts that the reaction rate (red arrow in Figure 4A) is approximately 3 times larger in the presence than in the absence of the allosteric modulator, if the transition state is located halfway on the reaction coordinate (*α* = 0.5).

To investigate the impact of *α* on the substrate uptake rate, we systematically varied the location of the transition state on the reaction coordinate. Figure 4B depicts the ligand-induced fold change (gain) of the substrate uptake velocity at different ligand concentrations as a function of *α*. As is evident from the plot, a ligand with the properties described in Figure 4A can accelerate the rate of substrate uptake by a maximum factor of 10. However, the magnitude of the gain depends on the value of *α* (i.e., the position of the transition state on the reaction coordinate) and the ambient ligand concentration. Notably, if *α* = 0, the modulator is unable to accelerate the reaction (i.e., gain = 1). In this context, it is important to emphasize that the position of the transition state on the reaction coordinate is an intrinsic property of a reaction and not subject to change. Thus, if and to which extent a candidate solute carrier is amendable to the desired allosteric action (e.g., acceleration of substrate uptake velocity), depends on the values of these parameters.

In Figures 4C, D we show the gain of the substrate uptake velocity for an allosteric ligand that is 100 times and 1000 times more selective for the OF state over the IF state, respectively as a function of *α* at different ligand concentrations: a more OF-selective ligand can be an even more effective positive allosteric modulator. The maximal gain at a saturating concentration of the allosteric ligand is 100 and 1000 in Figures 4C, D, respectively (cf., lines representing ≥0.3 mM in Figure 4C, and 1 M in Figure 4D).

In Figure 4E we plotted the normalized increase in substrate uptake velocity as a function of the concentration of the hypothetical OF-selective allosteric ligands considered in Figures 4B–D. It is evident from Figure 4E that there is a right shift of the EC_50_ value for the allosteric effect with increasing OF selectivity. This is because for the more selective allosteric ligands higher concentrations are required to afford occupancy of the ligand binding site in the low-affinity state (i.e., IF state). An OF selective allosteric ligands can only support the desired positive allosteric action, if it remains bound (i.e., it must not dissociate from the IF state).

This allows for the seemingly paradoxical situation, where - at a low ambient concentration of the allosteric ligand—a less selective modulator can afford a larger gain than a more selective one. This is shown in Figure 4F, where we assumed that the allosteric modulator was present at a concentration of 10 µM. In this instance, the more selective hypothetical allosteric modulators are less effective, if *α* = 0.5. With *α* = 1 (see Figure 4G), a moderate increase in ligand selectivity for the OF state increases the gain, but the relation is bell-shaped. Hence, in this case, there is also a loss in gain, if the hypothetical allosteric modulator is too selective for the outward-facing state OF. This is to say that the optimal selectivity for the OF state (i.e., the selectivity that gives rise to the largest gain) depends on the attainable modulator concentration. Because there are inherent limits in the (plasma) concentration, which can be realistically achieved, this consideration is relevant for the design of a therapeutically useful positive allosteric modulator.

In the simulations displayed in Figures 4B–G, we assumed that the conformational transition from the inward-to the outward-facing state (Figure 4A) always remained rate-limiting, regardless of the extent to which it was accelerated upon binding of the allosteric modulator. This, however, is not a realistic scenario: if the rate-limiting reaction in the transport cycle of a solute carrier is substantially accelerated, another reaction must eventually become rate-limiting. This was explored in the simulations shown in Figure 4H. Here, we assumed that the second slowest reaction was 10 times faster than the slowest reaction (i.e., 20 s^−1^ vs. 2 s^−1^) and that the allosteric modulators did not affect this second reaction. Plotted is the ligand-induced gain in the substrate uptake velocity as a function of *α*. Under these circumstances, there is little advantage in using a more state-selective modulator, even if it is applied at an unrealistic saturating concentration (e.g., 1 M).

### Negative allosteric modulators

**FIGURE 5:**
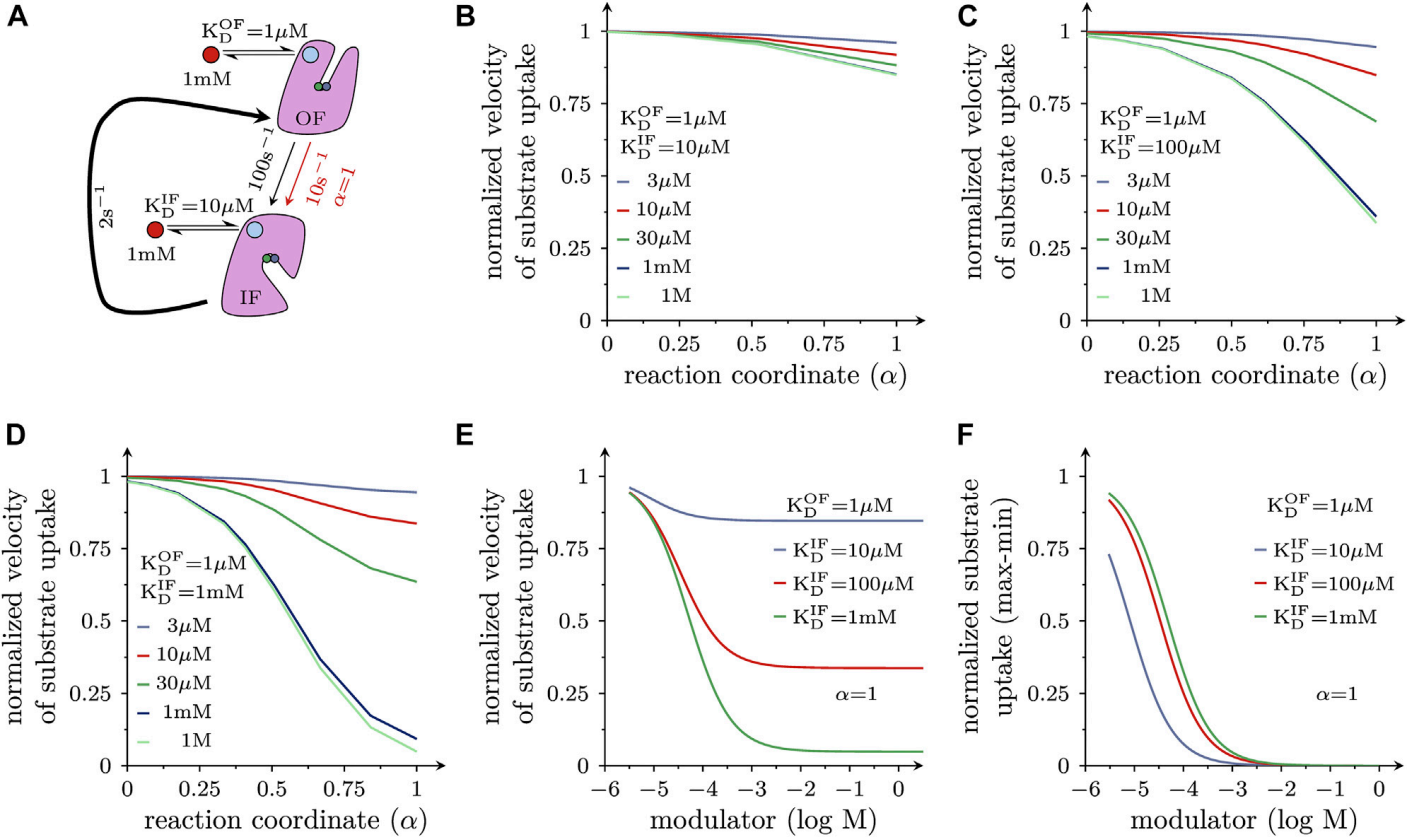
Negative allosteric modulators of solute carrier function **(A)** The scheme illustrates the reaction in the transport cycle of an SLC, which carries (co)-substrate through the membrane. This transition is a partial reaction in a forwardly biased reaction loop, because the transporter is assumed to operate under (ionic) conditions, which favor the forward cycling mode. In the absence of any (allosteric) ligand (black arrow), the rate of the transition equals 100 s^−1^. All other reactions in the loop—including the rate-limiting step—are represented by the bold curved arrow. It is further assumed that the hypothetical negative allosteric modulator binds to the substrate-bound outward-facing (OF) state with tenfold higher affinity than to the corresponding inward-facing (IF) state (with K_D_ = 1 µM and 10 μM, respectively). For *α* is 1 and in the presence of 1 mM of the modulator, the rate of the reaction dropped from 100 s^−1^ (black arrow) to 10 s^−1^ (red arrow). **(B)** Inhibition of substrate uptake by a negative allosteric modulator is plotted as a function of *α*. The concentrations of the negative allosteric modulator, which has affinities for substrate-bound OF and IF state as outlined in **(A)**, were varied over the indicated range (3 μM–1 M); velocity of substrate uptake is shown as normalized value (i.e., uptake in the absence of the allosteric modulator equals 1). **(C,D)** show the same plot as in **(B)** but for negative allosteric modulators, which are more selective for the substrate-bound OF-state (i.e., 100 fold and 1000 fold, respectively). The inhibition of substrate uptake velocity depends on *α* and on the selectivity of the allosteric modulator for the substrate-bound outward-facing (OF) state: it progressively increases as the selectivity of the modulator is raised from 10-fold **(B)** to 100-fold **(C)** and to 1000-fold **(D)**. **(E)** Plotted is the normalized substrate uptake velocity as a function of the concentration of hypothetical negative allosteric modulators, which differ in their selectivity for the substrate-bound OF-state (*α* = 1). Inhibition of substrate uptake increases with the selectivity of the modulator. Negative allosteric modulators allow for residual substrate uptake when present at a saturating concentration. **(F)** The data in E were normalized (1 = no inhibition, 0 = maximal inhibition) because differences in the IC_50_ values can be more readily appreciated in this representation. With increasing selectivity the IC_50_ value of the inhibitory effect is modestly shifted to higher modulator concentrations.

Allosteric modulators can also affect other reactions in the transport cycle of a solute carrier, e.g., the transition from the substrate-bound OF to the substrate-bound IF state. In fact, it can be argued that it is this reaction, which is targeted by the antidepressant drug vilazodone. Vilazodone is classified as a negative allosteric modulator of the serotonin transporter (SERT) based on its ability to inhibit 5-HT uptake in a non-competitive manner. A recent cryo-EM structure of SERT in complex with vilazodone revealed that this molecule bound to the S2 of SERT, rendering it likely that vilazodone and 5-HT can bind to this transporter simultaneously (Plenge et al., 2021). Thus, vilazodone is predicted to stabilize the substrate-bound OF state and to thereby slow the reaction, which carries (co)-substrate through the membrane. This reaction is depicted in the scheme shown in Figure 5A. The resulting model makes the following assumption: (i) this substrate translocation step is a partial reaction in a forwardly biased reaction loop and (ii) it proceeds at a rate, which is substantially higher than that of the slowest reaction. In the scheme of Figure 5A, the curved arrow represents all other reactions comprising the loop (including its rate-limiting step). Because the loop is biased into the forward direction the substrate-bound outward-facing (OF) state is the reactant state. Thus, in accordance with the considerations outlined above, a hypothetical allosteric modulator, which binds with higher affinity to the OF than to the IF state, is expected to reduce the rate of this reaction. For the simulation, we therefore first assumed that the substrate-bound outward- and inward-facing state bound the allosteric modulator with a K_D_ of 1 and 10 μM, respectively. LFER predicts that a modulator with these properties can reduce the rate of the considered reaction from 100 s^−1^ down to 10 s^−1^ if *α* = 1 (i.e., the transition state resembles the substrate-bound OF state). At lower values of *α*, the reduction is less pronounced. However, because a reaction rate of 10 s^−1^ is still considerably faster than that of the slowest reaction, the turnover rate (rate of the entire process) and thus, uptake velocity is only reduced by the modulator to a very modest extent. This is shown in Figure 5B where we plotted the inhibitory effect of a modulator with the properties described in Figure 5A on substrate uptake as a function of *α* for different modulator concentrations. The figure illustrates that a negative allosteric modulator, can allow for residual substrate uptake, even if it is present at a saturating concentration (≥1 mM; lines in magenta and red). Further enhancing the selectivity of the allosteric modulator for the outward-facing state to 100-fold (Figure 5C) or 1000-fold (Figure 5D) progressively reduces the level of residual substrate uptake. Figure 5E examines the concentration-dependent inhibition of substrate uptake by the three hypothetical negative allosteric modulators considered in Figures 5B–D for *α* = 1: the resulting concentration-response curves provide three insights: with increasing selectivity for the outward-facing state, (i) the extent of uptake inhibition is progressively augmented (Figure 5E) and (ii) the IC_50_ of the modulators shift modestly to the right (this can be readily appreciated from a replot of the data in Figure 5F; (iii) there is residual substrate uptake even at a saturating concentration of the most selective negative allosteric modulator (ratio K_D,OF_/K_D,IF_ = 1000; a green curve in Figure 5E).

### The allosteric modulation of a composite reaction can be described by an apparent *α* (α_app_)

**FIGURE 6:**
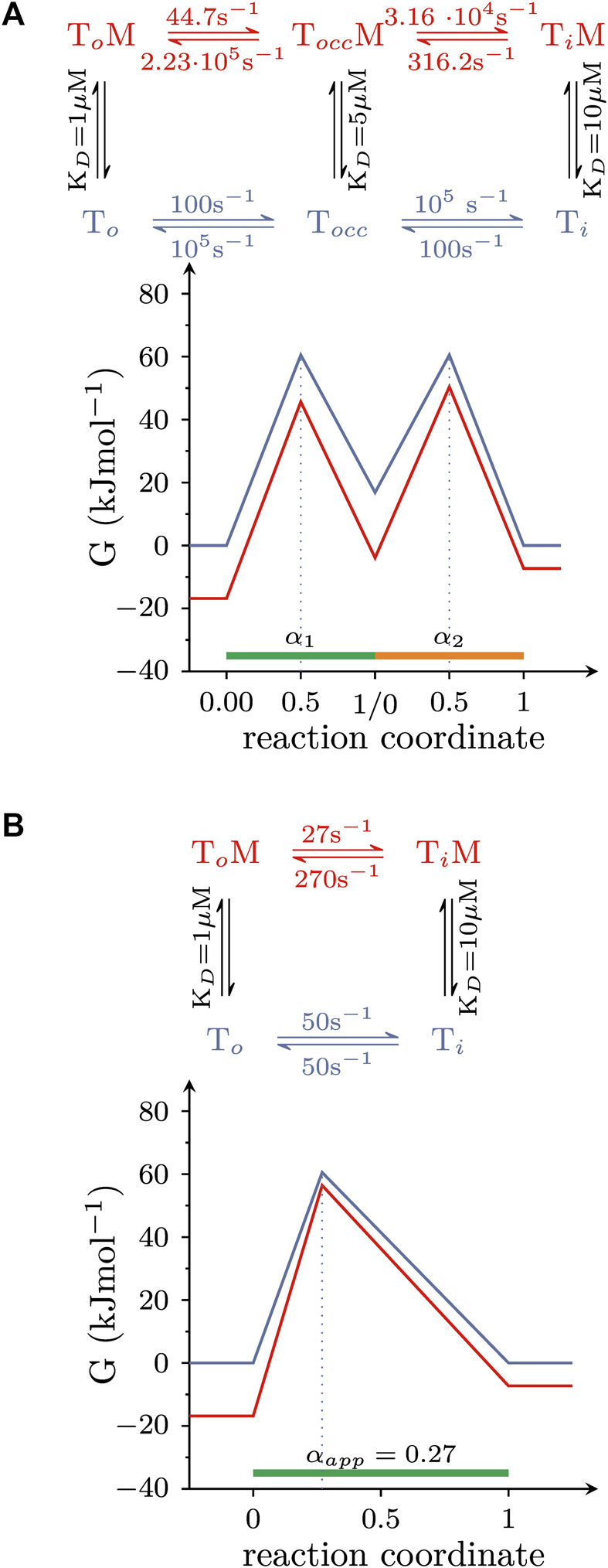
The amenability of a composite reaction by an allosteric modulator can be described by α_app_. **(A)** Hypothetical free energy landscape of the conformational rearrangement that carries (co)-substrate through the membrane and which surpasses a short-lived occluded state (T_Occ_). The reaction is assumed to be amenable by an allosteric modulator. Shown on top is the corresponding reaction diagram. **(B)** Equivalent free energy landscape of the reaction in **(A)**. The corresponding reaction scheme is shown on top. The scheme in **(A,B)** are indistinguishable in their ability to account for the (i) affinity of the modulator for the two stable ground states and (ii) the composite reaction rate both in the absence and presence of the modulator. These are the parameters, which can be measured in experiments.

In the simulations summarized above, we assumed that the conformational rearrangements, which the solute carriers underwent, were elementary reactions. This, however, is a simplification: when transitioning from outward-to inward-facing (or *vice versa*), transporters must visit short-lived intermediate states. One such intermediate is the occluded state, where the binding site of the substrate is sealed off on both sides. Accordingly, isomerization from the OF- to the IF state involves at least two elementary reactions: (i) the transition from the OF to the occluded and (ii) from the occluded to the IF state. Because each reaction entails a transition state, there are now two αs which need to be considered. However, the following example shows that the multiple αs of a composite reaction can be replaced by an apparent *α* (α_app_): Figure 6A illustrates a hypothetical landscape of the substrate translocation reaction, which also accounts for the occluded state. The corresponding reaction scheme is shown on top. The scheme makes the following assumptions: (i) the occluded state is short-lived (i.e., its lifetime is 10 microseconds); (ii) the value of *α* of each reaction (i.e., α_1_ and α_2_); is 0.5; (iii) the affinity of the intermediate state for the modulator lies between that of the two ground states, that is K_D_s of the (co)-substrate bound OF (T_O_ state in the scheme), occluded (T_OCC_), and IF conformation (T_I_)are 1, 5, and 10 μM, respectively. However, because the lifetime of the occluded state in Figure 6A is so short, it is not possible to resolve the reaction rates into and out of this state with any of the currently available methods. Observable quantities are the rate of the composite reaction in the presence and absence of the modulator and the affinity of the modulator for the two stable ground states (Hasenhuetl et al., 2015; Li et al., 2015; Bhat et al., 2023). These parameters allow for deducing the energy landscape shown in Figure 6B (the corresponding reaction scheme is shown on top). The schemes in Figures 6A, B are equivalent in their ability to account for the parameters, which are accessible to experimental determination. Thus, a value of 0.27 for α_app_ describes the overall reaction. This value of α_app_ remains a useful parameter to gauge the effect of an allosteric modulator, even if the reaction is composite. In the scheme outlined in Figure 6A, the transition states are located halfway between each pertinent reactant and product state. However, the (experimentally accessible) overall reaction shown in Figure 6B suggests that the transition state is closer to the reactant state. Thus, this comparison highlights the caveat that it is not possible to infer any structural resemblance of transition and ground states in a composite reaction (Huysmans et al., 2021). This can only be done, if the reaction is truly elementary.

## Discussion

Allosteric modulation of a drug target can be attractive for pharmacotherapy; this is exemplified by the success of benzodiazepines and related compounds, which are positive allosteric modulators of GABA_A_-receptors (Sigel and Ernst, 2018). Positive and negative allosteric modulation is not confined to cys-loop receptors and other ionotropic receptors: the calcium-sensing receptor is targeted by three approved positive allosteric modulators, cinacalcet, evocalcet, and etelcalcetide, which bind to distinct sites (Leach et al., 2020). Thus, allosteric modulation is worthwhile exploring. Transition state theory provides a theoretical framework to understand and predict the action of allosteric modulators. Here we focused on solute carriers, because they are attractive—albeit underdrugged—pharmacological targets (Wang et al., 2020; Casiraghi et al., 2021), their conformational cycle is well understood and thus amenable to kinetic modelling (Burtscher et al., 2018; Schicker et al., 2021; Schicker et al., 2022) and because allosteric modulators may remedy defects resulting from mutations (Bhat et al., 2021). We applied transition state theory by positing that—for partial reactions in the transport cycle of a solute carrier—a linear relation exists between the change in the free energy difference of reactant and product states ( $∆{∆G}_{R}$ ; $∆∆G_{P}$ ) and its change for the corresponding transitions states (∆∆ $G^{‡}$ ). This structure-reactivity relation is also known as LFER (linear free energy relationship). LFER is considered a semi-empirical rule on two grounds: (i) if specific reactions are subjected to experimental scrutiny, they are usually found to conform to LFER; (ii) for some simple reactions, it is possible to derive the underlying structure-reactivity relation from first principles. However, currently, there isn’t any stringent formulation of a general law, which would allow for deducing all observed LFERs (Leffler, 1963; Agmon, 1981).

LFER is not as fundamental as the law of energy preservation: this is evident from the observation that it was possible to violate LFER, while maintaining microscopic reversibility in the pertinent reaction schemes (see Figure 3). The position of the transition state on the reaction coordinate (*α*) is an important parameter in LFER. It is only possible to predict the effect of a given allosteric modulator, if *α* is known. Fortunately, experiments can be designed to determine the position of the transition state on the reaction coordinate. For this purpose, the reaction must be subjected to a perturbation that either increases or decreases its reaction rate. Partial reactions in the transport cycle of a solute carrier can be perturbed by (i) voltage changes (if the reaction is voltage-dependent), (ii) by mutations in the coding region of the candidate SLC gene (if this leads to a change in the reaction rate) and (iii) by the use of allosteric modulators. An example of such an analysis can be found in (Huysmans et al., 2021).

Monoamine transporters are of pharmacotherapeutic relevance and they are targets of popular, illicit drugs. Accordingly, the chemical space has been amply explored for possible ligands. In fact, several hundred ligands are known to bind to monoamine transporters (Sitte and Freissmuth, 2015): these include inhibitors, which bind to the orthosteric site, full and partial substrates (Hasenhuetl et al., 2019; Niello et al., 2020), negative allosteric modulators (Niello et al., 2020) and even an uncompetitive inhibitor (Bhat et al., 2023), but surprisingly, no positive allosteric modulator other than Zn^2+^ has been identified. Our analysis provides an explanation of why it is difficult to find positive allosteric modulators: A positive allosteric modulator must bind to the product state of the slowest reaction in the transport cycle of the targeted SLC with higher affinity than to the reactant state, but it must not be too selective. This requirement is difficult to fulfill. To the best of our knowledge, Zn^2+^ and related transitional metals are the only known example of a positive allosteric modulator in the entire superfamily of SLC transporters: Zn^2+^ and other transitional metals accelerate the transport cycle of the dopamine transporter (DAT) (Li et al., 2017). DAT harbors a Zn^2+^ binding site because evolution selected this transporter for allostery. It is, therefore, tempting to speculate that the adaptive search for optimizing the rate of substrate translocation shaped the binding site of Zn^2+^ such that this transition metal can afford a maximal increase in the dopamine uptake rate (Schicker et al., 2022). This notion is supported by the observation that Zn^2+^ is only modestly selective for the OF-state over the IF-state (i.e., the affinity of Zn^2+^ for the OF-state is approximately 10 times higher than for the IF-state). If Zn^2+^ were more selective for the OF-state, a considerably higher concentration of Zn^2+^ would be required to increase the substrate uptake rate. Yet, even if it were possible to reach this high Zn^2+^ concentration, little would be gained. This is because, in a realistic scenario, another reaction in the transport cycle inevitably becomes rate-limiting. As a consequence, the extra gain afforded by a more selective ligand does not translate into a higher substrate turnover rate. In DAT, this other reaction is predicted to be the transition that carries the substrate through the membrane. The rate of this reaction is about one order of magnitude higher than that of the rate-limiting reaction (i.e., the return step of the empty transporter from the OF to the IF state). A notable shortcoming of Zn^2+^ is that it does not discriminate between substrate-free and bound OF states. Therefore, Zn^2+^ also accelerates the return step from the substrate-bound IF to the corresponding OF state (Li et al., 2017). Accordingly, if the intracellular Na^+^ concentration (Na^+^_in_) is raised above 15 mM, Zn^2+^ inhibits substrate translocation (Li et al., 2015), because the substrate is released at a lower rate at elevated Na_in_^+^. This raises the abundance of the substrate-loaded IF state. As a consequence, the transporter more frequently returns in the substrate-bound form (i.e., it enters the exchange mode). Because Zn^2+^ accelerates this reaction it inhibits substrate uptake when Na_in_^+^ is high. It is possible to circumvent this issue with an allosteric ligand, which only binds to the substrate-free states. In this context, it is worth mentioning that we recently analyzed the effect of the antibody fragment 8B6 scFv on the transport cycle of SERT (Esendir et al., 2021). 8B6 scFv was produced to facilitate the crystallization of SERT (Coleman et al., 2016). Incidentally, 8B6 scFv was exquisitely selective for the substrate-free OF state over the substrate-bound OF state. This finding is encouraging because it shows that it ought to be possible to find ligands, which bind with higher affinity to substrate-free than to substrate-bound states. However, because 8B6 scFv had no appreciable affinity for the substrate-free IF state (i.e., it was too selective for the substrate-free OF state) it failed to act as a positive allosteric modulator (Esendir et al., 2021).

Another type of action is allosteric inhibition of substrate uptake. A hallmark of these compounds is their non-competitive mode of transport inhibition. To exert this effect, a negative allosteric modulator must bind with higher affinity to the reactant than to the product state of a partial reaction in the transport cycle of the candidate SLC. However, in contrast to a positive allosteric modulator, a negative allosteric modulator need not target the slowest reaction. In fact, the slowing of any reaction in the transport cycle is predicted to decrease substrate uptake. In addition, there isn’t any upper limit on the selectivity of such a molecule for the high-affinity state. A higher selectivity for the reactant state solely increases the efficiency of the modulator. The larger number of possible solutions, by which this type of action can be achieved, is presumably the reason why negative allosteric modulators are not as rare as their positive counterparts. A notable insight, which we gained from our analysis, is that a negative allosteric modulator can act as a partial inhibitor, if it is not too selective for the reactant state of the targeted reaction. This property can conceivably be of value for the treatment of a disease caused by malfunctioning SLCs. A scenario can be envisaged, in which it is beneficial to clamp substrate uptake to a given level: in contrast to orthosteric inhibitors, the action of allosteric inhibitors is not overcome by rising concentrations of substrate.

**FIGURE 7:**
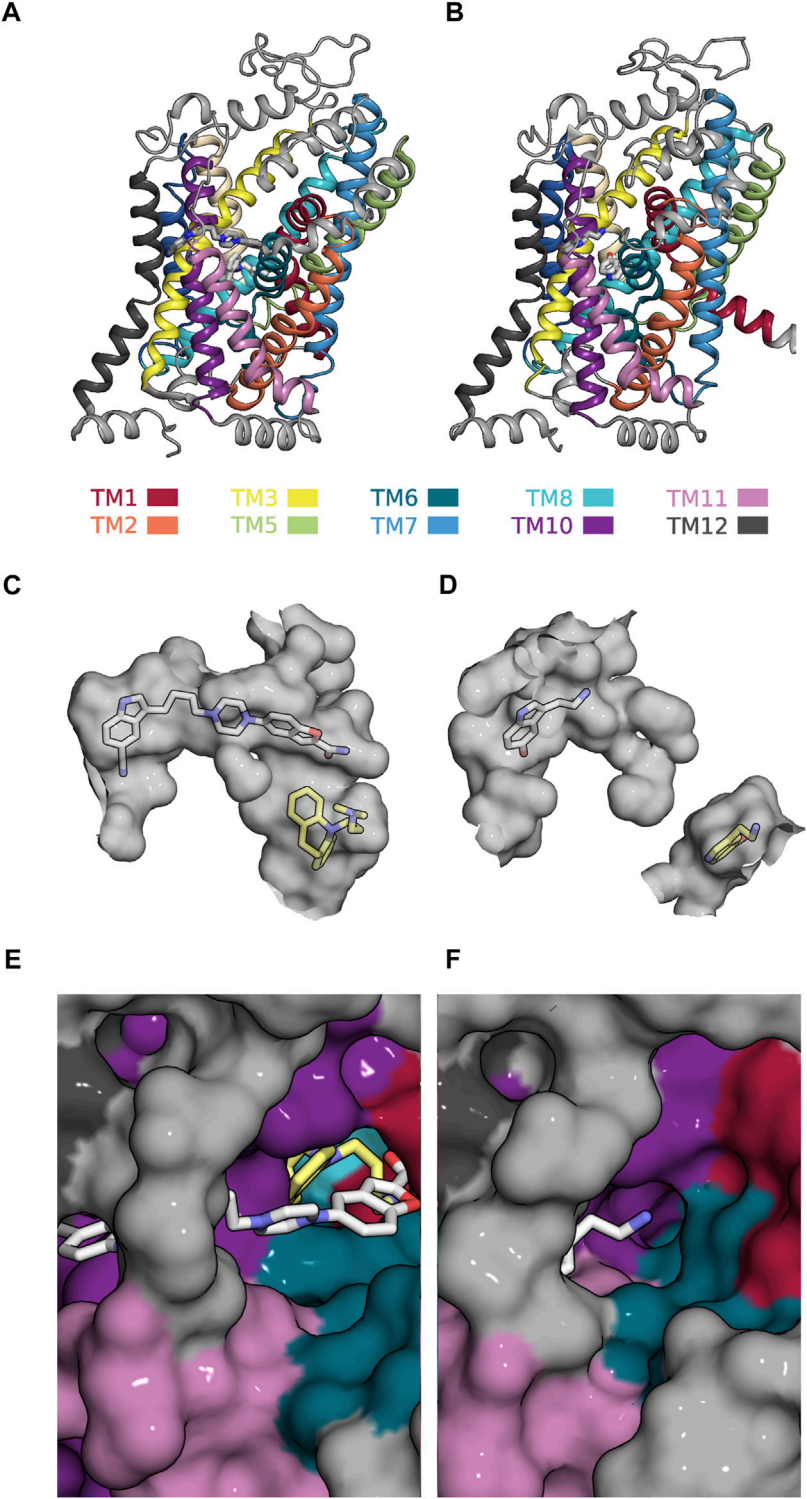
Allosteric binding site (i.e., the S2-site) in the outward- and inward-facing conformation of SERT **(A)** Side view onto the structure of SERT in the outward-facing conformation in complex with a vilazodone molecule bound to the S1- and another bound to the S2-site (PDB 7LWD) **(B)** Side view onto the structure of SERT in the substrate-bound inward-facing state, in which the S1- and S2-site are occupied with one 5-HT molecule each (PDB 7L19) **(C)** The accessible volume of the two binding pockets (S1-site and S2-site) in the outward-facing conformation. The bound vilazodone (grey carbon backbone) and imipramine molecules (yellow carbon backbone) reside in S2 and in S1, respectively. **(D)** The accessible volume of the S1- and the S2-site in the inward-facing conformation with the two bound 5-HT molecules visualized with yellow and grey carbon backbones, respectively. The S1-site is separated from the S2-site by the closed extracellular gate. **(E)** Top view onto the vilazodone molecule residing in the S2-site of the outward-facing state. The imipramine molecule residing in the S1-site is also visible. **(F)** Top view onto the 5-HT molecule bound to the S2-site of the inward-facing state.

Our analysis suggests that the most interesting modulatory actions (i.e., positive allosteric modulation and partial inhibition of SLC function) can be achieved by allosteric ligands, which are not too selective for either ground state of a partial reaction. The search for such compounds requires an adaption of the existing computational approaches. The present analysis provides some guidance for the design of the workflow, which ought to encompass the following steps: First, it is necessary to identify the reaction in the transport cycle of the candidate solute carrier, which is to be targeted to cause the desired effect. This, for instance, is the slowest reaction, if the goal is to find a positive allosteric modulator. Second, the structures of the corresponding reactant and product state must be searched for positions, which undergo a (modest) change. Third, (parallel) virtual screens must be conducted on these positions. The goal of this exercise is to find a ligand, which can bind to both ground states while assuming on each of them a unique binding mode. The proposed workflow can be illustrated by using SERT as an example: SERT harbors an allosteric binding site (i.e., the S2 site) to which vilazodone binds. A cryo-electron microscopy structure is available that shows vilazodone in complex with the outward open conformation of SERT (see Figure 7A). This structure corresponds to the reactant state of the reaction, which carries (co)-substrate through the membrane (cf. Figure 5). Incidentally, the structure of the corresponding product state (i.e., the substrate-bound inward-facing conformation) was also resolved (see Figure 7B) (Yang and Gouaux, 2021). In this structure, the S2 site is occupied by a second 5-HT molecule. Figures 7C, D depict the volumes (in grey) of the ortho- and allosteric binding pocket (i.e., S1 site and S2 site) in both states. In the inward-facing conformation these two binding sites are separated by the closed extracellular gate (Figure 7D). It is evident that the allosteric binding pocket is smaller in the inward-than in the outward-facing state such that the pose of vilazodone cannot be accommodated (cf. Figures 7C, D). Moreover, the path leading to the S2 site is narrower in the inward-facing conformation of SERT (Figure 7F) than in the outward-facing state (Figure 7F). This indicates a reduced accessibility to the S2 site upon transition to the inward-facing conformation. A computational search for a compound, which is not too selective for the reactant over the product state, is therefore proposed to encompass virtual screens in which each tested molecule is docked in parallel into the two binding pockets displayed in Figures 7C, D. In the selected example, only a molecule that fits into both binding pockets is likely to give rise to partial inhibition of substrate uptake. Full inhibition of substrate uptake was reported for vilazodone (Plenge et al., 2021). This is consistent with the observation that vilazodone cannot be accommodated by the S2 site of the product state (i.e., in the inward-facing conformation of SERT—cf. Figure 7D). However, it is conceivable that a slightly smaller molecule could fit into this pocket and thereby exert the desired modulatory action (i.e., partial inhibition of substrate uptake).

Another insight from our analysis is that the action of an allosteric modulator does not depend on the absolute affinities of the modulator for the reactant and product state but rather on their ratio. The ultimate goal of most docking campaigns is to find high-affinity ligands. Undoubtedly, high affinity is beneficial, because it limits off-target effects. However, we argue that the sole focus on high-affinity ligands is questionable: it may impede the discovery of allosteric ligands with useful properties.

## Data Availability

The original contributions presented in the study are included in the article/supplementary material, further inquiries can be directed to the corresponding author.
